# Drug-Initiated Activity Metabolomics Identifies Myristoylglycine as a Potent Endogenous Metabolite for Human Brown Fat Differentiation

**DOI:** 10.3390/metabo12080749

**Published:** 2022-08-16

**Authors:** Carlos Guijas, Andrew To, J. Rafael Montenegro-Burke, Xavier Domingo-Almenara, Zaida Alipio-Gloria, Bernard P. Kok, Enrique Saez, Nicole H. Alvarez, Kristen A. Johnson, Gary Siuzdak

**Affiliations:** 1 Scripps Center for Metabolomics, Scripps Research, La Jolla, CA 92037, USA; 2 California Institute for Biomedical Research (Calibr), Scripps Research, La Jolla, CA 92037, USA; 3 Department of Molecular Genetics, Donnelly Center, University of Toronto, Toronto, ON M5S 3E1, Canada; 4 Computational Metabolomics for Systems Biology Lab, Omics Sciences Unit, Eurecat—Technology Centre of Catalonia, 08005 Barcelona, Spain; 5 Department of Molecular Medicine, Scripps Research, La Jolla, CA 92037, USA; 6 Departments of Chemistry, Molecular, and Computational Biology, Scripps Research, La Jolla, CA 92037, USA

**Keywords:** metabolomics, activity metabolomics, myristoylglycine, obesity, brown adipose tissue, white adipose tissue, zafirlukast

## Abstract

Worldwide, obesity rates have doubled since the 1980s and in the USA alone, almost 40% of adults are obese, which is closely associated with a myriad of metabolic diseases such as type 2 diabetes and arteriosclerosis. Obesity is derived from an imbalance between energy intake and consumption, therefore balancing energy homeostasis is an attractive target for metabolic diseases. One therapeutic approach consists of increasing the number of brown-like adipocytes in the white adipose tissue (WAT). Whereas WAT stores excess energy, brown adipose tissue (BAT) can dissipate this energy overload in the form of heat, increasing energy expenditure and thus inhibiting metabolic diseases. To facilitate BAT production a high-throughput screening approach was developed on previously known drugs using human Simpson–Golabi–Behmel Syndrome (SGBS) preadipocytes. The screening allowed us to discover that zafirlukast, an FDA-approved small molecule drug commonly used to treat asthma, was able to differentiate adipocyte precursors and white-biased adipocytes into functional brown adipocytes. However, zafirlukast is toxic to human cells at higher dosages. Drug-Initiated Activity Metabolomics (DIAM) was used to investigate zafirlukast as a BAT inducer, and the endogenous metabolite myristoylglycine was then discovered to mimic the browning properties of zafirlukast without impacting cell viability. Myristoylglycine was found to be bio-synthesized upon zafirlukast treatment and was unique in inducing brown adipocyte differentiation, raising the possibility of using endogenous metabolites and bypassing the exogenous drugs to potentially alleviate disease, in this case, obesity and other related metabolic diseases.

## 1. Introduction

The prevalence of obesity has increased exponentially in the last 40 years worldwide, reaching pandemic level prevalence in the USA, as almost 40% of adults are considered obese [1]. A sustained obesity state is correlated with the development of metabolic syndrome-related diseases, such as type 2 diabetes, cardiovascular diseases, hepatic steatosis, and some types of cancer, increasing the morbidity and mortality of the population unless the obesity is reverted [2]. Obesity is manifested through an imbalance in energy homeostasis where the energy intake cannot be fully consumed by the body’s metabolism and energy expenditures. The non-consumed energy is stored mainly in form of triacylglycerol within the lipid droplets of white adipocytes in the white adipose tissue (WAT) [3].

Brown adipose tissue (BAT), a specialized tissue involved in thermogenesis, is an intriguing target for the treatment of metabolic syndrome-related diseases. Although brown adipocytes also store lipid droplets (normally of a greater number and of smaller size than white adipocytes), contrary to white adipocytes, brown adipocytes are composed of numerous mitochondria expressing uncoupling protein-1 (UCP1) in their inner membrane. This protein uncouples fatty acid oxidation from ATP synthesis by reversing the proton gradient, resulting in the dissipation of energy in the form of heat [4]. As a result, BAT activation leads to increased energy expenditure, contributes to glucose and lipid homeostasis, and potentially alleviates metabolic disease symptoms [3,4,5].

Since BAT-mediated thermoregulation is essential in the development and survival of mammalian newborns, large depots of BAT are normally found in human infants. Even though there is clear evidence of metabolically active BAT depots in adult humans, it is also known that BAT reservoirs decline with age and BMI [6,7]. In adult humans, two different types of brown adipocytes have been documented, the constitutive BAT adipocytes and the inducible brite (brown-in-white) adipocytes (also known as beige adipocytes), both expressing the protein UCP1. Brite adipocytes show a high degree of structural and functional similarity with brown adipocytes, although they present exclusive molecular signatures. The principal difference lies in the origin of brite adipocytes which are generated within pre-existing WAT upon cold exposure or stimulation of specific pathways, demonstrating the plasticity of the adipose organ [6,8,9]. Since constitutive BAT arises during embryogenesis and decays with age, the acquisition of a brite phenotype by white adipocytes (browning) is considered a promising approach to improving global metabolic health [8].

Brite adipocytes can be generated either from adipogenic progenitor cells or from differentiated white adipocytes in a reversible process. To date, several positive regulators of browning have been described in mouse models, including cold exposure, agonists of the beta-3-adrenergic receptor (ADRB3), and agonists of the PPAR-γ nuclear receptor [6,8]. These discoveries have directly impacted global metabolic health in diverse rodent models, as browning activation has been linked to reduced insulin resistance, loss of weight, and better control of hyperlipidemia [3,4,6,10]. However, the extrapolation of mice results into humans is still uncertain. Although a clear positive correlation between the presence of brown and brite adipocytes with leanness and insulin sensitivity has been documented in adult humans [3,4], the pharmacological approaches to enhance browning based on ADRB3 and PPAR-γ agonists have had no, or modest, success so far [3,4,8,9,10]. On one hand, ADRB3 expression in humans is lower compared to mice, and prolonged treatment with ADRB3 agonists results in the reduction in the endogenous activation of BAT [10]. On the other hand, PPAR-γ agonists, like thiazolidinediones, can induce functional brown adipocytes [11] and have proven effective in the treatment of insulin resistance in humans. However, experimental links between their role in browning and their insulin-sensitizing effect are scarce and some studies have demonstrated deleterious side effects following the administration of these drugs in patients [4,12].

Despite these unsuccessful attempts to find pharmacological modulators of adipocyte browning in humans, the demonstrated therapeutic potential of expanding brown adipose tissue in rodent models makes this approach an attractive target to battle obesity and other related diseases with high prevalence. Since in adult humans, UCP1-positive cells are predominantly of the brite type (coherent with the age-dependent decay of neonatal BAT) [13], it is worth exploring new therapeutic approaches that promote brite adipocyte differentiation. For example, the use of human Simpson–Golabi–Behmel Syndrome (SGBS) cells provides a unique tool to study adipocyte fate and differentiation in vitro. SBGS preadipocyte cells were derived from the stromal fraction of subcutaneous adipose tissue of a patient suffering SGBS [14]. SBGS preadipocytes can proliferate in vitro for many generations without being immortalized or transformed, overcoming the limited availability of adipose tissue to obtain primary preadipocytes for human studies [15]. Thus, SGBS cells are an excellent model to search for molecules capable of promoting brite adipocyte differentiation in humans.

To identify possible inducers of browning, we employed Drug-Initiated Activity Metabolomics (DIAM), an approach that merges exogenous drug metabolism and metabolomics [16,17]. After a high-throughput screening campaign using known small molecule drug libraries, we found that zafirlukast was able to induce the generation of functional brown adipocytes. Zafirlukast is an FDA-approved small molecule drug that has been commonly used for the prophylactic and chronic treatment of asthma for 25 years. This drug competitively blocks the cysteinyl leukotriene 1 receptor, preventing the potent pro-inflammatory endogenous lipid mediators cysteinyl leukotrienes from binding during the chronic inflammatory response in the airway [18]. This result was used to repurpose this drug and design an activity metabolomics set of experiments to identify endogenous metabolites involved in the induction of browning. The key finding was that myristoylglycine, an endogenous metabolite with no previously described biological activity, was synthesized upon adipocyte differentiation by zafirlukast. This metabolite was unique among other differentially regulated metabolites in that it promoted brown adipocyte differentiation when it was added to cells exogenously, without impacting cell viability. This study employs DIAM and introduces an endogenous inducer of brown adipocytes from adipocyte precursors in a human model and raises the possibility of the use of endogenous metabolites, typically safe and inexpensive, as a means to bypass treatment with drugs in metabolic syndrome-related diseases.

## 2. Results

### 2.1. High-Throughput Screening Reveals an Inducer of Brown Fat Differentiation

**Figure 1:**
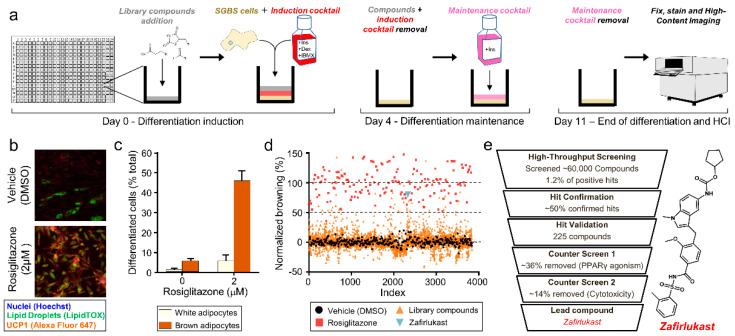
High-throughput screening reveals an inducer of brown fat differentiation. (**a**) Schematic of SGBS cells differentiation procedure. Human SGBS preadipocytes in media containing insulin, dexamethasone, and IBMX were plated directly on the compound in a 384 well-plate for induction. The induction media was then removed after 4 days and replaced with maintenance media containing only insulin for an additional 7 days. After 11 days, the plates were fixed, permeabilized, and stained for imaging. (**b**) SGBS treated with rosiglitazone served as positive controls for each plate. Cells were co-stained with anti-UCP1 antibodies (red), LipidTox (green) for lipid droplets, and Hoechst (blue) to visualize nuclei. (**c**) Quantification of rosiglitazone-induced brown adipocytes. Adipocytic differentiation rate was calculated based on an algorithm that identifies the area and intensity of lipid droplets and UCP1 staining. Brown adipocytes met a set threshold that contains lipid droplets in addition to UCP1 protein. Differentiated cells that have lipid droplets but no UCP1 were defined as white adipocytes. (**d**) Scatter plot representing a randomly selected batch of screening data (4000 of the total ~60,000 compounds screened). Each data point represents a single compound at 5 μM, normalized to the DMSO control (black). Rosiglitazone (red) at 2 μM served as a control. Zafirlukast is highlighted in light blue. (**e**) Schematic of preadipocyte screening and high-throughput workflow. Compounds were selected through hit confirmation and validation and then prioritized based on (1) bias for brown adipocytes versus white adipocytes, (2) lack of direct PPAR-γ agonism, and (3) lack of cytotoxicity. Zafirlukast matched our criteria for inducers of brown fat and became the basis of further investigation.

The acquisition of a brown phenotype in white adipose tissue adipocytes has been demonstrated as an effective approach to the alleviation of symptoms related to metabolic syndrome [3,4,10,12]. Thus, the principal goal was the design of a robust screening platform that allows for the analysis and identification of small molecules that induce brown adipocyte phenotype using high content imaging, as described in the Methods section. Unique to our screening was the use of human Simpson–Golabi–Behmel Syndrome preadipocyte cells (SGBS). Using a 384-well high-throughput screening format, SGBS cells were differentiated in the presence of induction media and compounds from the screening library. After 4 days of induction, cells were changed to maintenance media (containing insulin only) and continued to differentiate for an additional 7 days before they were fixed and stained for high-content imaging (Figure 1a). To better identify the compounds which could promote brown adipocyte differentiation from existing white adipocytes, the induction media (containing insulin, IBMX, and dexamethasone) was biased towards white adipocyte differentiation [19]. To accelerate the potential translation of these findings into human use, a focused set of ~12,000 FDA-approved therapeutics in a comprehensive library of high-value compounds (the ReFRAME collection), was initially chosen as a high priority. This library is composed of small molecule drugs which have already been clinically tested or registered for human use [20,21]. Additionally, ~48,000 compounds from other small molecule repositories including the Bioactive/LOPAC library, the Diversity Library from Life Chemicals, and the ChemDiv collection, were screened in parallel.

The thiazolidinedione drug rosiglitazone is a well-documented inductor of brown adipocyte differentiation through the activation of the peroxisome proliferator-activated receptor gamma (PPAR-γ) [4]. To validate the screening format and imaging parameters, rosiglitazone was used as a positive control to select active compounds. Through the imaging software, we gated the parameters to identify the total number of cells (by staining nuclei with Hoechst), the area and intensity of lipid droplets (by staining neutral lipids with LipidTox), and the UCP1 expression (using the UCP1 antibody conjugated with Alexa Fluor 647) (Figure 1b). Our algorithms then calculated the percentage of cells differentiated into white and brown adipocytes. Only cells containing lipid droplets and UCP1 were considered brown adipocytes. Those cells which contained lipid droplets but do not express UCP1 were defined as white adipocytes. Rosiglitazone was able to differentiate 51.9% of SGBS preadipocytes into adipocytes, where 46.1% were brown adipocytes and 5.8% were white adipocytes. On the other hand, treatment with induction media alone produced 5.7% brown adipocytes and 1.5% white adipocytes, resulting in only 7.2% total differentiation and demonstrating that induction media alone does not promote brown adipocyte differentiation (Figure 1c).

Using this model, a collection of ~60,000 compounds from multiple libraries was screened [22]. The summarized result of a particular primary screening batch containing ~4000 compounds is shown in Figure 1d. The percentage of differentiated brown adipocytes for each compound was normalized considering 100% to be the differentiation induced by rosiglitazone and 0% to be the differentiation induced by DMSO (vehicle). A hit is considered when a compound is able to induce a normalized differentiation of 50% of the initial preadipocytes into brown adipocytes. From the primary screening, 1.2% of compounds were hits (Figure 1e). Hits were confirmed and validated through a concentration–response titration curve. Only 225 candidates remained after these steps. Since PPAR-γ agonists have been demonstrated to increase cardiovascular risk in patients [12], candidates were counter-screened using a PPAR-γ competitive binding assay to rule out any agonist of this receptor. Finally, the cytotoxicity of compounds was evaluated using CellTiter-Glo assays to remove compounds that were cytotoxic (Figure 1e).

Through this screening, several compounds that promoted SGBS preadipocyte differentiation into brown adipocytes were identified. Among them, we found epinephrine, a classic ADRB3 agonist [23], and a subset of lysophosphatidic acid (LPA) receptor antagonists. Most importantly, we identified zafirlukast, a synthetic antagonist of the cysteinyl leukotriene receptor 1 (CysLTR1) commonly used to treat asthma [18], which was able to differentiate SBGS into brown adipocytes to a similar extent to the positive control rosiglitazone (Figure 1d,e).

### 2.2. Zafirlukast Induces Brown Adipogenesis in Human Preadipocytes

**Figure 2:**
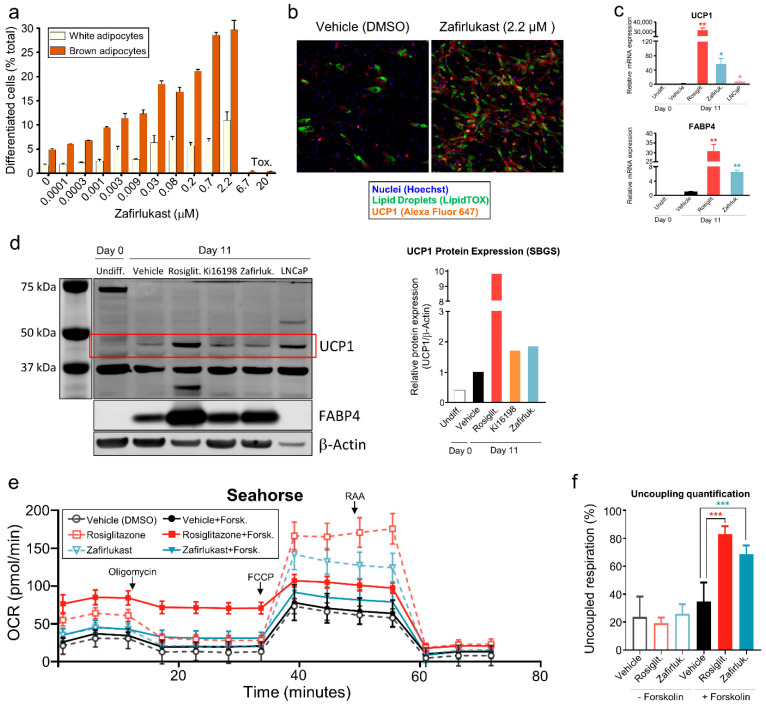
Zafirlukast induces metabolically active brown adipocytes. (**a**) Quantification of the dose–response effect of zafirlukast in brown adipocyte differentiation based on HCI data (**b**) High-content imaging of differentiated SGBS cells treated with 2.2 μM zafirlukast. Fixed cells were stained with LipidTox (green) for neutral lipid droplets, anti-UCP1 antibodies (red), and Hoechst (blue) to visualize nuclei. (**c**) Gene expression of markers for brown adipocytes (UCP1) and adipogenesis (FABP4) in zafirlukast-treated cells, measured by qPCR. (**d**) Western blot analysis and quantification of UCP1 and FABP4 in SBGS cells treated with rosiglitazone, Ki16198 (LPA antagonist), and zafirlukast. LNCaP prostate cancer cell line was used as a positive control for UCP1 since it is known to express this protein. (**e**) Cellular respiration was analyzed using an XF96 extracellular flux analyzer as described in Methods. Oxygen consumption rate of zafirlukast and rosiglitazone over time was measured by the interference of mitochondrial pathways with specific inhibitors in the absence or presence of forskolin as a surrogate measure of cAMP-induced uncoupled respiration. (**f**) Quantification of uncoupled respiration. (**c**,**f**) *** *p* < 0.001; ** *p* < 0.01; * *p* < 0.05, one-way ANOVA.

To further evaluate the browning properties of zafirlukast, a dose–response curve of differentiation was carried out. Unsurprisingly, it was observed that zafirlukast increased brown adipocyte differentiation in a dose-dependent manner (Figure 2a). Although this compound passed the cytotoxicity counter-screening carried out at lower concentrations, brown adipocyte differentiation could not be evaluated at higher doses due to cell viability declining (see below). The formation of lipid-filled and UCP1-expressing brown adipocytes can be clearly observed in Figure 2b. Cells treated with vehicle barely showed staining with either lipid droplet of UCP1 markers.

To confirm the assignment of a brown phenotype in zafirlukast-treated cells, qPCR and Western blotting were performed to measure gene and protein expression of pivotal markers of adipocyte differentiation. At day 11 of differentiation, the mRNA of the brown adipocyte marker UCP1 was increased by ~55-fold in zafirlukast-treated cells compared to control (Figure 2c). Total adipocyte differentiation was assessed by fatty acid binding protein 4 (FABP4) expression [24], which showed a ~7-fold increase (Figure 2c). Next, we investigated if UCP1 mRNA expression was observed in parallel with protein synthesis. Immunoblotting revealed that zafirlukast treatment increased UCP1 protein levels in SGBS adipocytes (Figure 2d). Quantitation and normalization of band intensity indicated a ~9-fold increase in rosiglitazone-treated cells. Likewise, UCP1 levels were doubled by zafirlukast treatment (Figure 2d). LnCAP, a cancer stem cell line, was used as a positive control of UCP1 expression [25].

### 2.3. Differentiation of Preadipocytes by Zafirlukast Creates Metabolically Active Adipocytes

We next ascertained the metabolic activity of differentiated SGBS beige adipocytes in the presence of zafirlukast using the Seahorse mitochondrial stress test. This test reveals key parameters of metabolic function through sequential injections of modulators of the electron transport chain. Maximal mitochondrial oxidative capacity (measured after injecting the ionophore FCCP) was increased in zafirlukast-treated cells (Figure 2e). Next, we acutely treated these cells with forskolin as a surrogate measure of cAMP-induced uncoupled respiration. Uncoupled respiration was measured by determining the difference in oxygen consumption rates basally compared to post-oligomycin injection (ATP synthase inhibition). We found that the basal OCR in the zafirlukast-treated cells is greatly increased compared to vehicle-treated cells, leading to a 2.2-fold increase in mitochondrial uncoupling (Figure 2e,f), similar to forskolin. These data demonstrate that zafirlukast can induce functional cells that acquire a similar phenotype to mature beige adipocytes, capable of uncoupling mitochondrial respiration from ATP synthesis to produce heat.

Altogether, we discovered that the presence of zafirlukast in the white adipocyte-biased differentiation media induces brown adipocytes from SGBS preadipocytes. These differentiated cells show the phenotypic markers of mature brown adipocytes and were demonstrated to efficiently uncouple mitochondrial respiration from ATP synthesis.

### 2.4. Metabolomics Screenings for Endogenous Modulators of Brown Adipocyte Differentiation

The demonstrated ability of zafirlukast to induce functional human brown adipocytes, together with the concerns about its cytotoxicity at high doses led us to design a drug-initiated activity metabolomics (DIAM) screening platform with a dual goal: finding endogenous molecules that could directly promote adipocyte browning and obtaining mechanistic insight into how zafirlukast leads to brown adipocyte differentiation. The proximity of metabolomics with phenotypic outcomes, together with its ability to characterize most small molecule mediators that impact biochemical processes, makes this technology amenable to discovering endogenous metabolites that impact phenotype [16,17]. Prior to designing the metabolomics screening, we interrogated whether the effect of zafirlukast on browning was mediated by its known antagonism on the CysLTR1 receptor.

**Figure 3:**
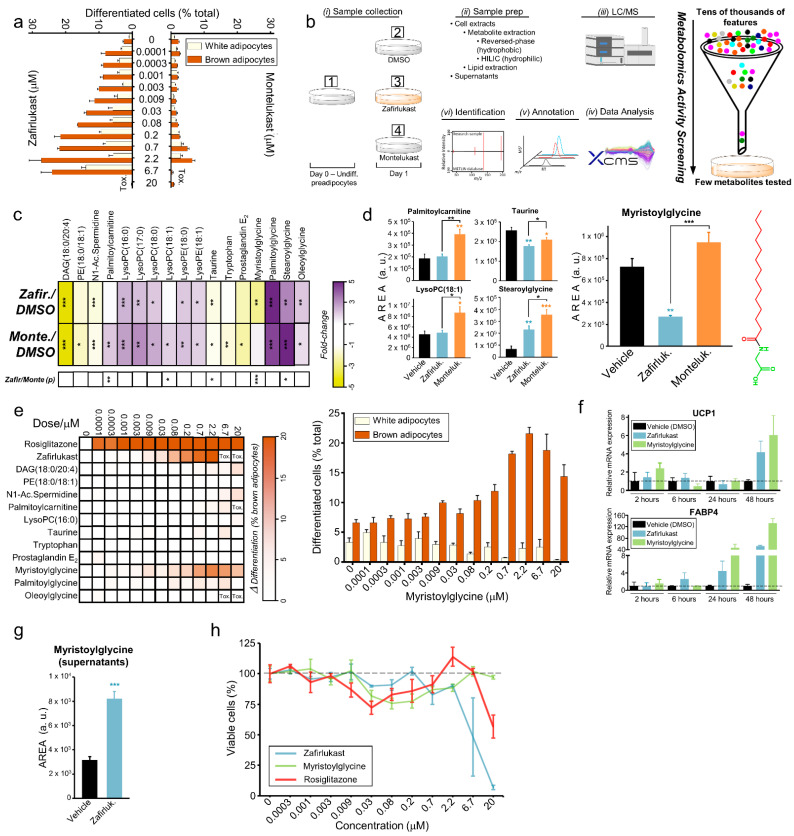
Drug-Initiated Activity Metabolomics (DIAM) screening allows for the discovery of myristoylglycine, which recapitulates brown adipocyte differentiation. (**a**) Comparative dose–response effect of zafirlukast and montelukast in brown adipocyte differentiation based on HCI data. (**b**) Activity metabolomics screening experimental design. Four parallel untargeted metabolomics and lipidomics analyses were carried out by liquid chromatography coupled with high-resolution mass spectrometry. Raw data were processed by XCMS Online, annotated, and statistically significant features were fragmented for identification. This workflow allowed a drastic reduction in data complexity. From more than 30,000 initial annotated features, only 17 metabolites were prioritized and screened for activity in brown adipocyte differentiation. (**c**) Fold-change representation using DMSO as a reference of the 17 metabolites changed between treatments at day 1. Fold-change was calculated using the metabolite abundance (average integrated area under the curve) for each treatment. (**d**) Bar plots of the 5 metabolites that were statistically significantly changed between zafirlukast and montelukast treatments. Myristoylglycine's chemical structure is plotted next to the graphs. (**e**) Dose–response effect of the metabolites prioritized in brown adipocyte differentiation based on HCI data. Rosiglitazone and zafirlukast are the positive controls of differentiation. LysoPC(16:0) was chosen as representative of the 6 lysolipids. Stearoylglycine was not commercially available. The dose–response of myristoylglycine in brown and white adipocyte differentiation is shown enlarged. (**f**) Gene expression of markers for brown adipocytes (UCP1) and adipogenesis (FABP4) in myristoylglycine-treated cells, measured by qPCR. Zafirlukast was included as the positive control. (**g**) Bar plot of myristoylglycine measured in the supernatants of the vehicle and zafirlukast-treated cells at day 1 of differentiation. (**h**) Cell viability dose–response of myristoylglycine, zafirlukast, and rosiglitazone evaluated using CellTiter-Glo luminescent cell viability assay following the instructions of the manufacturer. (**c**,**d**,**g**) * *p* < 0.05, ** *p* < 0.01, *** *p* < 0.001, one-way ANOVA followed by a Tukey’s honestly significant difference *posthoc* test. N = 6 biological replicates per treatment.

For this reason, we evaluated whether montelukast, an analogous CysLTR1 antagonist [26], had a similar effect on brown adipocyte differentiation. Montelukast did not induce brown adipocyte differentiation, indicating that zafirlukast does not promote browning by its antagonism of the CysLTR1 (Figure 3a). Moreover, montelukast is an excellent control to isolate the metabolic changes produced by zafirlukast during brown adipocyte differentiation from those produced by its known antagonism of the CysLTR1 receptor.

Taking this into account, we designed an activity metabolomics screening workflow with four experimental groups (Figure 3b). We compared (1) untreated SGBS cells at day 0 of differentiation (undifferentiated preadipocytes), (2) SGBS cells treated with DMSO for 24 h (vehicle), (3) SGBS cells treated with zafirlukast for 24 h (cells determined to differentiate into brown adipocytes), and (4) SGBS cells treated with montelukast for 24 h (control of CysLTR1 antagonism) (Figure 3b). The choice of t = 24 h to evaluate the metabolic changes was based upon preliminary experiments that showed that most of the transcriptomic changes were produced during very early differentiation. Both cell extracts and supernatants were processed by different analytical platforms, allowing for an ample covering of the metabolome, including the lipidome (Figure 3b).

Raw mass spectrometry data were processed for a multigroup analysis using XCMS Online [27]. After annotation [28,29], only strongly statistically significant molecular features (q < 0.01, one-way ANOVA followed by a local false discovery rate correction) were subjected to tandem MS experiments for their identification. 37 different molecules were identified in this first screening (Figure 3b). In order to prioritize metabolites that would be eventually tested for brown adipocyte differentiation, those that were present in the differentiation media or the treatments were ruled out. Additionally, those compounds whose changes were attributable to differentiation media only (*p* > 0.05, one-way ANOVA only considering treatments at 24 h) were also discarded. After filtering out these compounds, only 17 endogenous metabolites showed changes ascribable to zafirlukast and/or montelukast treatment (Figure 3c). Overall, the qualitative response of these metabolites to both drugs is similar. However, there were five metabolites significantly altered between both drug treatments (*p* < 0.05, Tukey’s honestly significant difference *posthoc* test), demonstrating that most of the metabolic responses induced by zafirlukast are unspecific and/or due to its antagonism of the CysLTR1 receptor (Figure 3c,d). Among those five candidates, only myristoylglycine levels were altered by zafirlukast treatment (cells committed to differentiate into brown adipocytes) but remained unchanged by montelukast treatment (which induces a similar phenotype to the vehicle) (Figure 3d).

### 2.5. Myristoylglycine Recapitulates Brown Adipocyte Differentiation

Similar to the high-throughput screening experiments, SGBS preadipocytes were treated for the first 4 days of differentiation with the metabolites together with the induction cocktail (biased toward white adipocyte differentiation). After an additional 7 days in maintenance media, cells were stained and quantified for lipid droplets and UCP1 (Figure 1a). Rosiglitazone and Zafirlukast were used as positive controls. LysoPC (16:0) was chosen as representative of the 6 lysolipids [30,31,32]. Stearoylglycine standard was not commercially available. Among all screened endogenous molecules, only myristoylglycine was able to induce brown adipocyte differentiation without promoting differentiation of preadipocytes into white adipocytes (Figure 3e). Of note, palmitoylglycine or oleoylglycine, structurally similar compounds to myristoylglycine, did not induce any differentiation. To confirm the acquisition of a brown phenotype by myristoylglycine-treated cells, gene expression of UCP1 was measured. An early UCP1 gene expression was induced by myristoylglycine, indicating that the molecular machinery for the acquisition of a brown phenotype is activated at the early stages of differentiation. Total adipocyte differentiation, assessed by FABP4 expression, was also enhanced by myristoylglycine treatment (Figure 3f).

The observation that intracellular myristoylglycine decreased in zafirlukast-induced brown adipocytes (Figure 3d) may appear to be contradictory to the evidence that the external addition of this metabolite to preadipocytes promotes their differentiation. To shed light on this, the metabolite levels were measured in the supernatants, observing that extracellular myristoylglycine increased in zafirlukast-induced brown adipocytes compared to control cells (Figure 3g). This change was not found in the first untargeted experiments because the peak intensity was below the established threshold. However, the other three acylglycines discovered in the screening (palmitoylglycine, stearoylglycine, and oleoylglycine) that were all intracellularly increased in zafirlukast-treated cells (and montelukast-treated cells) (Figure 3c), were not found in the supernatants. This suggests that myristoylglycine could be synthesized and secreted by SGBS cells during their differentiation into brown adipocytes.

One of the objectives of discovering endogenous inducers of browning is bypassing the use of drugs to achieve the desired phenotype. This is of special interest in this model since zafirlukast promoted cell toxicity at concentrations higher than 2.2 µM in the screening experiments (Figure 2a and Figure 3a,e). For this reason, we evaluated cell viability under the treatment with various browning agents. Myristoylglycine did not impact cell viability at concentrations up to 20 µM. Conversely, zafirlukast was toxic at both low and high concentrations, similar to rosiglitazone (Figure 3h).

In summary, by designing a drug-initiated activity metabolomics screening we were able to prioritize 17 endogenous candidates to be tested for browning activity from the initial over 30,000 annotated metabolic features. Among them, only myristoylglycine, a lipidated amino acid with no activity described up until now [33], promoted brown adipocyte differentiation without impacting cell viability.

### 2.6. Myristoylglycine Is the Only Lipidated Amino Acid That Induces Browning

**Figure 4:**
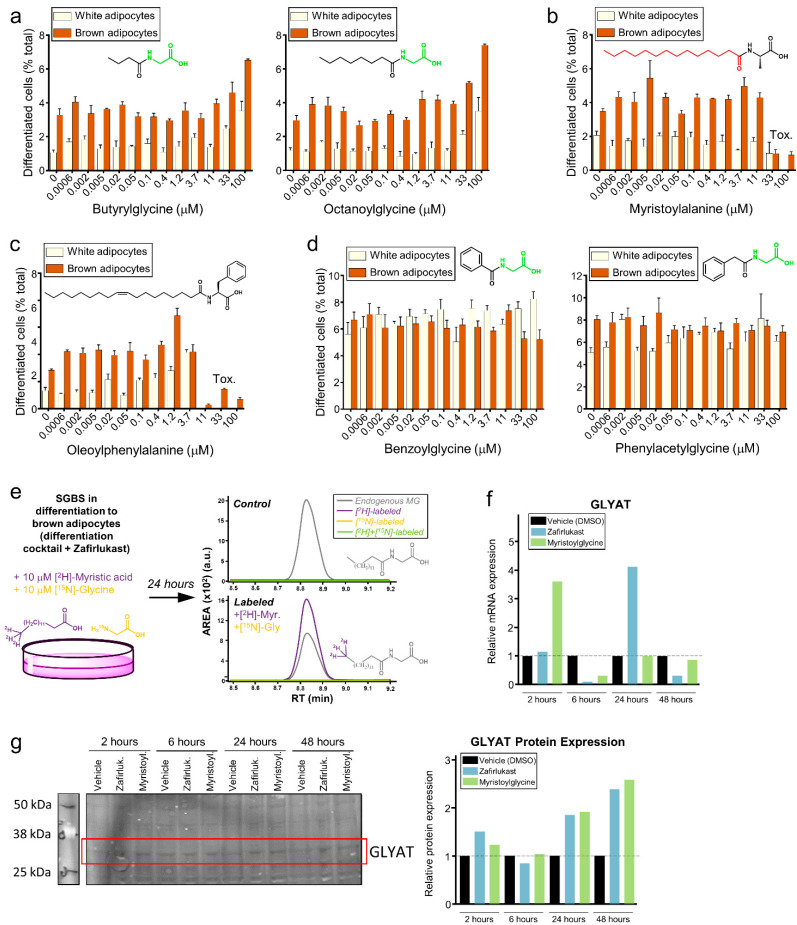
DIAM identified myristoylglycine is synthesized during browning, being the only lapidated amino acid that induces brown adipocyte differentiation. (**a**–**d**) Dose–response effect of butyrylglycine and octanoylglycine (**a**), myristoyl alanine (**b**), oleoylphenylalanine, (**c**) and benzoylglycine and phenylacetylglycine (**d**) in brown adipocyte differentiation based on HCI data. (**e**) SGBS cells were labeled with 10 mM of [^2^H]-Myristic acid and [^15^N]-Glycine and allowed to differentiate with zafirlukast for 24 h. Afterward, metabolites were extracted and labeled and myristoylglycine was measured by LC/MS. De novo synthesis of myristoylglycine at the expense of labeled myristic acid was found during brown adipocyte differentiation by zafirlukast. (**f**) Human Glycine N-Acyltransferase (GLYAT) gene expression measured by qPCR at different time points. (**g**) Western blot analysis and quantification of human GLYAT in SGBS cells treated with zafirlukast at different time points.

Of all observed acylglycines (myristoylglycine (14:0), palmitoylglycine (16:0), stearoylglycine (18:0), and oleoylglycine (18:1)), myristoylglycine possesses the shortest fatty acyl chain. Thus, we evaluated whether the myristoylglycine-driven enhancement of brown adipocyte differentiation compared to the others might be due to its lower molecular mass and hydrophobicity. To test this hypothesis, cells were treated with butyrylglycine (a short-chain acylglycine) and octanoylglycine (a medium-chain acylglycine). None of these molecules induced a significant differentiation of preadipocytes into brown adipocytes, even at concentrations as high as 100 µM (Figure 4a), suggesting that the differential activity of myristoylglycine compared to longer-chain acylglycines cannot be attributed to these biophysical differences.

Free fatty acids (FFA) are well-known regulators of adipocyte differentiation and function. The regulation of their intracellular levels in brown adipocytes is pivotal for thermogenesis through multiple mechanisms [34,35,36,37]. Four FFAs were among the 37 highly dysregulated metabolites in the metabolomics screening, including myristic acid. However, myristic acid was not tested in the activity screening since its regulation was only dependent on the presence of the differentiation media, regardless of the drugs (Figure 3b and Figure S1a). For this reason, we explored whether the metabolic regulation of myristoylglycine during brown adipocyte differentiation could be driven by its hydrolysis products (myristic acid and glycine). First, free glycine levels in cells were measured under the same four experimental conditions used in the metabolomics screening, but no differences were observed (Figure S1a). Similarly, we determined free myristic acid in the supernatants. Extracellular myristic acid levels dropped sharply with the use of the differentiation media, similarly to intracellular myristic acid (Figure S1a). 

Even though myristic acid regulation does not appear to be involved in brown adipocyte differentiation, the decrease observed between undifferentiated cells and cells differentiated for 24 h, raised the possibility that this FFA was essential to form other myristoylated amino acids with a role in adipocyte differentiation, similar to myristoylglycine. We searched for the presence of nine other myristoylated amino acids (myristoyl-Phe, -Ala, -Val, -Leu, -Gln, -Lys, -Ser, -Trp, and -Tyr) in the original data sets, but none were found in the cell extracts or supernatants. Afterward, we tested the only commercially available compound, myristoyl alanine, which did not induce any differentiation and was toxic at high concentrations (Figure 4b).

On the other hand, it has been recently described that brown adipocytes can synthesize a vast number of lipidated amino acids from FFAs and free amino acids in the extracellular milieu through the secreted enzyme peptidase M20 domain-containing 1 (PM20D1). These lipidated amino acids directly bind mitochondria, acting as endogenous respiration uncouplers [38,39,40]. The fact that several fatty acids are dysregulated at day 1 of differentiation raised the possibility that the lipidated amino acids found in that study could play a role in SGBS cell differentiation into brown adipocytes. One of them was oleoylglycine [38], which did not induce browning in our model (Figure 3e). Additionally, we tested N-oleoylphenylalanine for brown adipocyte differentiation. Although this is one of the most potent endogenous uncouplers discovered [38], N-oleoylphenylalanine did not produce any significant differentiation to brown adipocytes and was toxic at concentrations higher than 3.7 µM (Figure 4c).

Surprisingly, two more conjugation products of glycine with organics acids were discovered in the first screening: benzoylglycine and phenylacetylglycine. Both metabolites were found in the supernatants and their levels dramatically dropped after one day of differentiation, regardless of the presence of the drugs (Figure S1b). We also tested these metabolites for possible browning activity, but similar to the previous experiments, benzoylglycine and phenylacetylglycine did not induce any differentiation when they were exogenously added to cells (Figure 4d).

Overall, we demonstrated that even though other structurally related molecules are dysregulated during adipocyte differentiation, myristoylglycine was the only endogenous metabolite able to promote preadipocyte differentiation into brown adipocytes effectively.

### 2.7. Myristoylglycine Is Synthesized during Brown Adipocyte Differentiation

Myristoylglycine-induced browning was not mimicked by several acylglycines with multiple fatty acyl lengths, other lipidated amino acids, or other glycine conjugation compounds. In addition, although some related metabolites appeared dysregulated in the metabolomics screening, myristoylglycine was the only metabolite that changed in the preadipocytes determined to differentiate into brown adipocytes (zafirlukast-treated) compared to DMSO- and montelukast-treated cells (Figure 3b–d). Finally, myristoylglycine was found to increase in the supernatants of cells committed to differentiate into brown adipocytes (Figure 3g). Altogether, these data generated the hypothesis that myristoylglycine synthesis and accumulation in the extracellular media could be actively regulated during brown adipocyte differentiation of SGBS cells.

To study the in vivo synthesis of myristoylglycine, SGBS preadipocytes were labeled with [^2^H]-Myristic acid and [^15^N]-Glycine and allowed to differentiate into brown adipocytes for 24 h (Figure 4e). Afterward, endogenous myristoylglycine, [^2^H]-Myristoylglycine (only labeled with myristic acid), [^15^N]-Myristoylglycine (only labeled with glycine), and [^2^H,^15^N]-Myristoylglycine (labeled with both myristic acid and glycine) were measured. It was observed that this metabolite was synthesized De Novo during brown adipocyte differentiation relying on exogenous myristic acid but using endogenous glycine as substrates (Figure 4e).

Zafirlukast addition to SBGS cells has been demonstrated to promote changes in up to four acylglycines, as well as to induce the De Novo synthesis and secretion of myristoylglycine. The synthesis of acylglycines is primarily carried out by the enzyme glycine N-acyltransferase (GLYAT), a mitochondrial enzyme highly expressed in kidney, liver, and adipose tissue by humans [41,42]. Thus, the gene expression of GLYAT in zafirlukast-treated cells was measured by qPCR. Zafirlukast induced a four-fold increase in GLYAT mRNA, peaking at 24 h of treatment (Figure 4f) and suggesting that myristoylglycine synthesis and activity on browning may be related to zafirlukast. Afterward, the protein expression was measured, indicating that GLYAT protein is increased in zafirlukast-treated cells at 24- and 48-h post-treatment (Figure 4g), suggesting that myristoylglycine regulation during brown adipocyte differentiation promoted by zafirlukast might not be coincidental and that the synthesis of this metabolite may represent an important mechanism by which zafirlukast promotes browning.

In summary, we observed that the treatment of cells with zafirlukast promotes the synthesis of myristoylglycine via GLYAT activation. Browning of SGBS cells by zafirlukast might be mediated, at least in part, by myristoylglycine, whose addition to the cells suffices to recapitulate the effect of the drug in brown adipocyte differentiation without impacting cell viability.

## 3. Discussion

Trans-differentiation of adipogenic precursors into brown adipocytes is recognized as a fascinating strategy to improve global metabolic health. Expansion of the brown adipose tissue increases energy expenditure, relieves dyslipidemia, and regulates blood glucose concentration, alleviating the causal symptoms of metabolic syndrome-related diseases [3,4,5,8,10]. In this research, we designed a high-throughput screening platform to screen for drugs that promoted brown adipocyte differentiation from human preadipocyte precursors (SGBS cells) in the presence of a white-biased adipocyte differentiation media. To streamline the potential translation of this discovery, we prioritized the use of the ReFRAME collection [20,21]. In this library of FDA-approved drugs, the time-consuming effort to optimize the molecules for clinical use through structure–activity relationship has already been completed, thus the process to tune molecules to a specific application is much shorter with minimal cost. Zafirlukast was found to effectively promote the differentiation of SGBS cells, creating metabolically active cells able to uncouple respiration from energy production that present the phenotypic features of mature brown adipocytes.

Zafirlukast is a well-characterized antagonist of the CysLTR1 receptor commonly prescribed for the treatment of the symptoms of asthma by blocking the cysteinyl leukotrienes triggered bronchoconstriction [18]. In these experiments, we discovered that zafirlukast is toxic for SGBS preadipocytes at concentrations higher than 2 µM and that its mechanism of action on adipocyte browning was different from the CysLTR1 antagonism since montelukast, an analogous drug, was unable to induce any differentiation. For these reasons, an activity metabolomics screening platform was designed to find close metabolic signatures to zafirlukast-induced brown adipocyte differentiation. The use of montelukast as a negative control of differentiation was essential to separate the metabolic changes produced by blocking the CysLTR1 receptor during the differentiation from those ascribable to the acquisition of a brown phenotype.

One of the most exciting applications of untargeted metabolomics is the discovery and prioritization of endogenous metabolites to be used as modulators of disease (reviewed in [16,17]). The screening performed here is a strong example, showing the impact that the activity metabolomics approach may have in biomedical sciences. Through a simple experimental design (four experimental conditions comparatively analyzed by four analytical platforms), we were able to first reduce the initial ~30,000 annotated features to 37 identified metabolites. Of those 37, only myristoylglycine changed in cells committed to differentiate into brown adipocytes (zafirlukast-treated) compared to vehicle, without being modified in cells treated with an analogous drug that induced a comparable phenotype to vehicle (montelukast). Attending to this evidence, myristoylglycine appeared to be the closest metabolic signature to brown adipocyte differentiation triggered by a repurposed use of zafirlukast, which is isolated from CysLTR1 blockage.

Unsurprisingly, myristoylglycine induced brown adipocyte differentiation to a similar extent to zafirlukast, an effect that was not mimicked by any of the other 11 metabolites initially tested. Different representative combinations of amino acids and fatty acyl chains, some among the 37 identified metabolites during the metabolomics screening, were also tested without showing any impact on brown adipocyte differentiation. Since the beige phenotype in white adipose tissue is flexible and reversible, the functional activation of brown adipocytes requires the concomitant expression of the thermogenic program along with the stimulation of the uncoupling response [4,10]. Oleoylphenylalanine and oleoylglycine have been demonstrated as effective inductors of respiration uncoupling in mature brown adipocytes highly expressing UCP1 already [38,39]. However, these metabolites did not have any effect on brown adipocyte differentiation. Whether myristoylglycine can trigger respiration uncoupling remains to be determined, but it is tempting to speculate that these structurally and functionally related lipidated amino acids could have a coordinated role in the differentiation of brown adipocytes expressing UCP1 and in the further activation of the thermogenic machinery, finally leading to respiration uncoupling.

The De Novo synthesis of myristoylglycine during brown adipocyte differentiation was observed to use (at least in part) exogenously added myristic acid. However, labeled glycine was not found as part of the newly synthesized molecule. This might be explained because of the different basal concentrations of both components of myristoylglycine: while the intracellular concentration of myristic acid in cells is lower than 1 mM, glycine was measured up to 1 mM concentrations (Figure S1a), therefore, the added glycine (10 mM) is readily diluted in the endogenous pool, making the detection of possible incorporation into De Novo synthesized myristoylglycine very challenging, hence the three orders of magnitude difference between myristic acid and glycine endogenous concentration would explain the outcome of the labeling experiments.

Multiple indications pointed to a tight connection between zafirlukast-induced brown adipocyte differentiation and the regulation of myristoylglycine levels. The strongest indication was the fact that of the thousands of metabolic features measured in the comprehensive untargeted metabolomics analysis, only myristoylglycine was changed upon induction of brown adipocyte differentiation by zafirlukast without being altered by the negative control montelukast. Other evidence includes the selectivity of this metabolite to promote browning compared with structurally similar compounds, the higher intensity of myristoylglycine compared to palmitoylglycine, stearoylglycine, and oleoylglycine, even when palmitic, stearic, and oleic acid concentration is significantly higher than myristic acid or the presence of the metabolite in the supernatants of zafirlukast-treated cells. This suggests that myristoylglycine may have potent signaling properties in terms of specificity and efficacy, triggering specific pathways involved in the activation of the brown adipocyte metabolic machinery. Moreover, the fact that the myristoylglycine is actively synthesized and secreted into the supernatants indicates that it may be acting on an autocrine or paracrine pathway, with the potential to be supplemented exogenously to induce this differentiation in more complex models.

In follow-up experiments, it was discovered that zafirlukast promotes the synthesis of myristoylglycine through the activation of its synthesizing enzyme GLYAT. While there are several enzymes capable of glycine N-acyltransferase activity that are able to catalyze the synthesis of acylglycines from acyl-CoA and glycine, GLYAT is the most effective described yet [42,43,44,45,46]. This enzyme is highly expressed by humans in kidney, liver, and adipose tissue [41,42]. While the expression in the kidney and liver is related to its well-known role in the detoxification of organic acids such as benzoic acid and other acidic compounds of xenobiotic origin [45,47], the expression of this enzyme in adipose tissue has barely been explored. Here, we provide evidence that preadipocytes in differentiation express GLYAT and that zafirlukast promotes its overexpression at the gene and protein level. GLYAT overexpression has been observed in the adipose tissue of obesity-resistant mice compared to mice that develop obesity in response to a high-fat diet, together with increased urinary excretion of isovalerylglycine and hexanoylglycine [48,49]. This evidence points towards a role of adipose GLYAT and secreted acylglycines in the development of an adipose-resistant phenotype in an in vivo mice model of obesity, similar to the results observed in our in vitro human model.

Altogether, we demonstrated the ability of drug-initiated activity metabolomics to discover metabolites that can bypass the use of pharmaceuticals in the induction of certain phenotypes. We also showed that it is possible to use endogenous metabolite drug combinations to increase the effectiveness of a treatment, as has been demonstrated with taurine-miconazole as well as inosine-pranobex combinations [50,51]. The continued interest in BAT [52,53] and the past and current success of these experiments, make this combination approach even more attractive.

## 4. Conclusions

In summary, despite the challenges associated with the discovery of effective and safe inducers of BAT from mouse models that are ineffective at accurately mimicking human metabolism, and the significant side effects of pharmaceutical drugs, there is continued interest in the generation of BAT [52,53]. To facilitate the discovery of safe and effective BAT inducers, we employed a Drug-Initiated Activity Metabolomics (DIAM) approach that uses a three-step process, the first step relied on human cell lines and employed a high throughput screening approach to initially identify a BAT inducer (or drug), the pharmaceutical repurposed drug, zafirlukast. Specifically, BAT production was examined using a high-throughput screening approach using human Simpson–Golabi–Behmel Syndrome (SGBS) preadipocytes designed for the discovery of the repurposed drugs. Zafirlukast was identified in these screenings as an effective compound for functional brown adipocyte differentiation from adipocyte precursors. This human model was also found to be successful in the presence of a white-biased adipocyte differentiation media.

The second step applied activity metabolomics to identify differentially regulated endogenous metabolites upon zafirlukast-facilitated BAT induction. The metabolomics approach enabled the prioritization of 17 candidates to be tested for browning activity from the initially observed 30,000 plus annotated metabolic features. 

The final third step involved testing the prioritized 17 endogenous metabolites for BAT induction. Myristoylglycine, a lipidated amino acid with no previously identified activity [33], mimicked the browning properties of zafirlukast. Myristoylglycine was also found, unlike zafirlukast, to not impact cell viability. Further to this, using isotopically labeled substrates, myristoylglycine was found to be bio-synthesized upon zafirlukast treatment, thus indicating that zafirlukast activity may be related to its ability to produce myristoylglycine. DIAM helped discover myristoylglycine as a unique promoter of brown adipocyte differentiation, without impacting cell viability, raising the possibility of using endogenous metabolite(s) to alleviate metabolic diseases. Overall, DIAM represents an unconventional alternative drug discovery approach, that relies on a greater understanding of exogenous drug metabolism to discover endogenous metabolites that can effectively act on a disease state

## 5. Materials and Methods

### 5.1. Cell Culture

Human Simpson–Golabi–Behmel syndrome (SGBS) preadipocyte cells were kindly provided by Dr. Martin Wabitsch [14,15]. SGBS preadipocytes were grown at 37 °C (5% CO_2_) in SGBS Base Medium (containing DMEM/F12 medium, 3.3 mM biotin, 1.7 mM pantothenate, and 1% antibiotic-antimycotic) supplemented with 10% fetal bovine serum. 

Upon passaging, cells were trypsinized, collected, and spun down at 1250× *g* for 5 min at 20 °C to pellet. Cells were then resuspended in growth media and seeded at an optimal density to maintain 75–80% confluency before passaging every 3–4 days.

### 5.2. Induction and Differentiation of Adipocytes

For SGBS, compounds at the indicated concentrations were spotted onto 384-well black clear bottom plates (Greiner, Monroe, NC, USA). Negative control wells consisted of DMSO while positive controls contained 2 µM of Rosiglitazone (Cayman Chemical, Ann Arbor, MI, USA). SGBS cells in induction media (SGBS Base Medium with 10 μg/mL Insulin, 0.5 mM 3-isobutyl-1-methylxanthine (IBMX), and 1 μM Dexamethasone) were seeded directly on top of compound at a concentration of 1.1 × 10^5^ cells/mL (~5500 cells/well). After 4 days of induction, the media was changed to a maintenance medium (SGBS Base Medium with 10 μg/mL Insulin). Every 2–4 days afterward, the media was again exchanged for maintenance media until day 11.

### 5.3. Immunofluorescence Staining

For UCP1 staining, adherent adipocytes were fixed with 5% paraformaldehyde for 10 min at room temperature (RT), followed by permeabilization with 1% Triton X-100 for another 10 min at RT. Cells were then blocked using Blocking Buffer 1 (2% BSA and 2.5% goat serum) and incubated for 1 h at RT. Next, plates were incubated with primary antibody for 16 h at 4 °C. Primary antibody containing UCP1 (Sigma U6382, 1:100) was diluted in Blocking Buffer 2 (2% BSA).

For MitoTracker staining, 25 nM of MitoTracker was added onto live cells and incubated at 37 °C for 30 min. After staining, cells were washed 2 times with fresh media and then fixed with 5% paraformaldehyde for 10 min at RT. 

For both types of staining, plates were washed 3 times with 1X PBS and secondary antibody was added. Secondary antibody containing Hoechst (1:6000) for nuclei staining, Alexa Fluor 647 goat Anti-Rabbit (1:1000) (for UCP1 staining only), and HCS LipidTOX Green Neutral Lipid Stain (Life Technologies, Carlsbad, CA, USA, 1:800) were diluted in Blocking Buffer 2. After 1 h of incubation at room temperature, plates were washed 3 times with 1X PBS and left in 1X PBS to be imaged.

### 5.4. Imaging

Plates were imaged using the CellInsight CX5 High Content Screening (HCS) Platform (Thermo Fisher Scientific, Waltham, MA, USA). Parameters were set to first identify individual cells by detection of Hoechst if they met a certain intensity and area. The software was then programmed to capture the total and average intensity of UCP1 and LipidTOX signals based on nuclei identification. These values were then used to set thresholds of events measuring the total percentage of adipocytes, percentage of white adipocytes, and percentage of brown adipocytes. Cells that contained both UCP1 and LipidTox staining were defined as brown adipocytes. Cells that contained only LipidTox were defined as white adipocytes. The sum of brown and white adipocytes was defined as total adipocytes. The total cell number was detected using Hoechst.

### 5.5. Counter Screening Assays

PPAR-γ agonism was checked using the LanthaScreen TR-FRET PPAR-γ competitive binding assay kit (Thermo Fisher Scientific). Manufacturer protocol was followed.

Cell toxicity was assessed using the CellTiter-Glo luminescent cell viability assay (Promega, Fitchburg, WI, USA). Cells were plated at 5500 cells/well in the presence of compound and regular growth media and incubated at 37 °C for 48 h. Manufacturer protocol was followed.

### 5.6. RNA Isolation, cDNA Synthesis, and Quantitative RT-PCR

RNA was isolated using the Qiagen RNeasy mini kit following manufacturer protocol. The concentration of eluted RNA was determined using Nanodrop. 20 ng of RNA was reversely transcribed into cDNA using Quanta qScript SXLT cDNA supermix. qPCR was done in triplicates on the ViiA 7 real-time PCR system (Thermo Fisher) using SYBR advantage qPCR premix (Clontech).

Oligonucleotides for human gene expression studies were as follows: FABP4, 5′-TGCAGCTTCCTTCTCACCTT-3′ (sense) and 5′-GGCAAAGCCCACTCCTACTT-3′ (antisense); HPRT, 5′TGACACTGGCAAAACAATGCA-3′ (sense) and 5′-GGTCCTTTTCACCAGCAAGCT-3′ (antisense) and 5′UCP1, 5′-CCAACTGTGCAATGAAAGTGT-3′ (sense) and 5′-CAAGTCGCAA-GAAGGAAGGTA-3′(antisense). All primers were custom ordered from Integrated DNA Technology.

### 5.7. Western Blotting

Cells were lysed using RIPA Buffer (Thermo Fisher Scientific) supplemented with protease inhibitor cocktail set I (EMD Millipore, Burlington, MA, USA). Protein samples were separated on 4–20% SDS-PAGE (Thermo Fisher Scientific) and transferred for 90 min at 40 V onto PVDF membranes (EMD Millipore). Membranes were blocked using Odyssey blocking buffer (Licor). Membranes were incubated with primary antibodies at 4 °C overnight then washed with PBS-Tween and incubated with secondary antibodies for 1 h at room temperature. Western blots were imaged and quantitated using the Odyssey infrared imaging system. Western blot analysis was carried out using the following antibodies: UCP1 (Abcam ab155117, 1:500), FABP4 (Abcam ab66682, 1:1000), and β-actin (Santa Cruz sc-47778, 1:1000).

### 5.8. Seahorse

SGBS preadipocytes were plated at a concentration of 1 × 10^5^ cells/mL (15,000 cells/well) in gelatin-coated XFe96-well cell culture microplates (Agilent Technologies, Santa Clara, CA, USA). At confluence, cells were treated with compounds and differentiated into adipocytes as described above. Day 11 adipocytes were incubated with XF base medium (Agilent Technologies) containing 2 mM Glutamine, 1 mM sodium pyruvate, and 18 mM glucose for 1 h. The oxygen consumption rate (OCR) from adipocytes was measured using the XFe96 Seahorse flux analyzer through sequential injections of 2 μM oligomycin, 1 μM FCCP (carbonyl cyanide-4(trifluoromethoxy)phenylhydrazone), and 0.5 μM RAA (rotenone and antimycin A). For acute forskolin treatment, 10 μM of forskolin was added during the incubation with XF base medium for one hour prior to running the Seahorse assay.

### 5.9. Metabolite Extraction for Mass Spectrometry

Extraction of metabolites from cell extracts: Polar and slightly non-polar metabolites were extracted from cell extracts as described elsewhere [54]. Briefly, 1 mL of cold acetonitrile/methanol/water (2:2:1, by vol.) was added to SGBS cell extracts. Cells underwent 3 cycles of freeze/thawing to precipitate protein. Each cycle included a flash freezing step in liquid nitrogen for 1 min, vortexing for 30 s, and sonication in an ice-cold bath for 15 min. Afterward, cells were incubated at −20 °C for 60 min. Finally, proteins were pelleted by centrifuging samples at 16,000× *g* for 15 min at 4 °C. Supernatants containing all metabolites were transferred to another tube and dried down, while the precipitated protein amount was measured by the BCA method [55]. Finally, metabolite extracts were reconstituted with acetonitrile/water (1:1, *vol*/*vol*) prior to their analysis by liquid chromatography/mass spectrometry (LC-MS). Metabolite extracts were vortexed, sonicated, and centrifugated to precipitate non-soluble molecules. The volume of acetonitrile/water was adjusted using the protein concentration of each sample, to use the same amount of starting material for the untargeted metabolomics analyses.

Extraction of lipids from cell extracts: Total lipid fraction was isolated using the method of Bligh and Dyer [56]. Briefly, cell extracts were dissolved in 200 µL of water. 750 µL of chloroform/methanol 1:2 (*vol*/*vol*) were added afterward, and cells were vortexed for 90 s. Later, 250 µL of chloroform and 250 µL of water were added. Cells were vortexed for 90 s and centrifuged at 8000× *g*, at 4 °C for 5 min. The organic layer (bottom) was transferred to a new vial and dried down. Finally, lipids were reconstituted with acetonitrile/2-propanol (1:1, *vol*/*vol*) prior to the lipidomics analysis by LC-MS. In order to normalize the results, the volume of reconstitution solvent was also calculated taking into account the protein content of each sample.

Extraction of metabolites from supernatants: Metabolites secreted in supernatants were extracted by solid-phase extraction (SPE) using Bond Elut C18-OH columns (Agilent Technologies). Before starting the extraction, 10% (*v*/*v*) of methanol and 0.5% (*v*/*v*) of acetic acid were added to the supernatants. First, SPE columns were conditioned with 3 mL of methanol and 3 mL of water. Afterward, samples were loaded into the columns. Before eluting the metabolites, non-specific bound compounds to the columns were washed with 3 mL of 10% methanol in water (*v*/*v*). Finally, metabolites were eluted with 1 mL of pure methanol and recovered in Eppendorf tubes. Finally, metabolites were dried down and reconstituted with acetonitrile/water (1:1, *vol*/*vol*) prior to their analysis by liquid chromatography/mass spectrometry (LC-MS). To normalize the results, the volume of reconstitution solvent was also calculated taking into account the protein content of each sample.

### 5.10. Untargeted Metabolomics

Metabolites extracted from cells were analyzed both by reversed-phase (RP) and hydrophilic interaction liquid chromatography (HILIC) to cover the widest portion of the metabolome [54]. Since the SPE column used to extract metabolites from supernatants is suitable for slightly hydrophobic compounds, metabolites from supernatants were only analyzed by reversed-phase chromatography.

Both RP and HILIC analyses were carried out in a Bruker Impact II quadrupole/time-of-flight (q-ToF) mass spectrometer coupled to a Bruker Elute UHPLC (Bruker, Billerica, MA, USA). Data were acquired over an *m*/*z* range of 50–1000 Da. The electrospray source conditions were as follows: end plate offset = 500 V, dry gas temperature = 200 °C, drying gas = 6 L/min, nebulizer = 1.6 bar, and capillary voltage = 3500 V. 

The same mobile phases were used for both RP and HILIC chromatography, consisting of 0.1% formic acid in water (*v*/*v*) as phase A and 0.1% formic acid in acetonitrile (*v*/*v*) as phase B. The flow through the column in both cases was 150 µL/min. An ACQUITY BEH C18 column (1.0 × 100 mm, 1.7 µm, Water Corporation, Milford, MA, USA) was used for the RP analysis, and an ACQUITY BEH Amide (1.0 × 100 mm, 1.7 μm, Water Corporation, Milford, MA, USA) was used for the HILIC analysis. The gradient for RP chromatography consisted of 99% A for 1 min, 1% A over 9 min, and held at 1% A for an additional 3 min. The gradient for HILIC consisted of 1% A for 1 min, 35% A over 13 min, 60% A over 3 min, and held at 60% A for 1 additional minute. Data were acquired in positive ion mode. For identification purposes, putative molecules of interest were fragmented at 3 different collision energies (10, 20, and 40 eV). 

### 5.11. Untargeted Lipidomics

Lipids were profiled by RP chromatography in positive ion mode, using the method described herein [57] with minor modifications. The UHPLC system and MS setup were the same used for the metabolomics analyses. The phase A consisted of water/acetonitrile (4:6, *v*/*v*) with 0.1% formic acid (*v*/*v*) and 1 mM ammonium formate, while the phase B was composed of 2-propanol/acetonitrile (9:1, *v*/*v*) with 0.1% formic acid (*v*/*v*) and 1 mM ammonium formate. An ACQUITY BEH C18 column (1.0 × 100 mm, 1.7 µm, Water Corporation, Milford, MA) was used. The gradient consisted of 68% A for 1 min, 55% A over 1 min, 48% A over 0.5 min, 42% A over 1.5 min, 34% A over 1.5 min, 30% A over 1.5 min, 25% A over 2 min, 3% A over 1.5 min, and held at 3% A for an additional 2 min. The flow through the column was 150 µL/min. For identification purposes, putative molecules of interest were fragmented at 3 different collision energies (10, 20, and 40 eV).

### 5.12. Isotope Labeling

SGBS preadipocytes were labeled with 10 mM [^2^H]-Myristic acid and [^15^N]-Glycine and allowed to differentiate into brown adipocytes for 24 h. Endogenous myristoylglycine, [^2^H]-Myristoylglycine (only labeled with myristic acid), [^15^N]-Myristoylglycine (only labeled with glycine), and [^2^H,^15^N]-Myristoylglycine (labeled with myristic acid and glycine) were monitored by LC/MS/MS using the analytical approach described in the untargeted metabolomics section.

### 5.13. Data Analysis

Raw.d data files were converted to. mzXML format using ProteoWizard MS Converter version 3.0.7529 [58]. Data were processed using XCMS Online (https://xcmsonline.scripps.edu) (accessed on 28 June 2017) [27]. Peaks were first detected, aligned across samples, and integrated. Then, the features (a set of integrated peaks with a particular *m*/*z* and retention time) underwent isotope removal and adduct and common losses annotation [28]. Finally, after the statistical analysis, only the features with q < 0.05 were selected for identification via MS/MS experiments. To identify these metabolites, the resulting MS/MS spectra were matched with the 870K METLIN database [59].

### 5.14. Statistics

For untargeted metabolomics and lipidomics experiments, 6 biological replicates per treatment were used. Data were expressed as mean ± SEM. Multigroup statistical significance of each feature was automatically determined by XCMS Online [27] using a one-way ANOVA followed by a local false discovery rate (FDR) correction of the *p*-values to generate the corresponding q-values. Features were considered statistically significant when the false discovery rate was q < 0.05. Once metabolites were identified, for the pairwise comparison, a Tukey’s honestly significant difference (HSD) posthoc test (parametric) was carried out. Metabolites were considered statistically significant between DMSO and zafirlukast/montelukast or between montelukast and zafirlukast when *p* < 0.05. For high-throughput screening, 3 biological replicates were used. Data were expressed as mean ± SEM. Statistical comparison was carried out using a one-way ANOVA, considering statistically significant differences with *p* < 0.05. Molecular biology (WB, qPCR, and Seahorse) experiments were independently repeated at least three times with incubations in triplicate and the data are expressed as mean ± SEM. Statistical analysis was carried out by one-way ANOVA, taken as statistically significant differences with *p* < 0.05.

## Data Availability

The data presented in this study are available in article and Supplementary Material.

## Supplementary Materials

The following supporting information can be downloaded at: https://www.mdpi.com/article/10.3390/metabo12080749/s1. Figure S1. Bar plots of myristoylglycine-related metabolites during SBGS cells differentiation in the presence of zafirlukast and montelukast.
