# Supplementary material for: Drug-Initiated Activity Metabolomics Identifies Myristoylglycine as a Potent Endogenous Metabolite for Human Brown Fat Differentiation
Source: Metabolites. 2022 Aug 16;12(8):749. doi: 10.3390/metabo12080749 (PMC9415469; doi:10.3390/metabo12080749)
Supplement: Supplementary file 1 [file metabolites-12-00749-s001.zip › metabolites-1848791-supplementary.pdf]

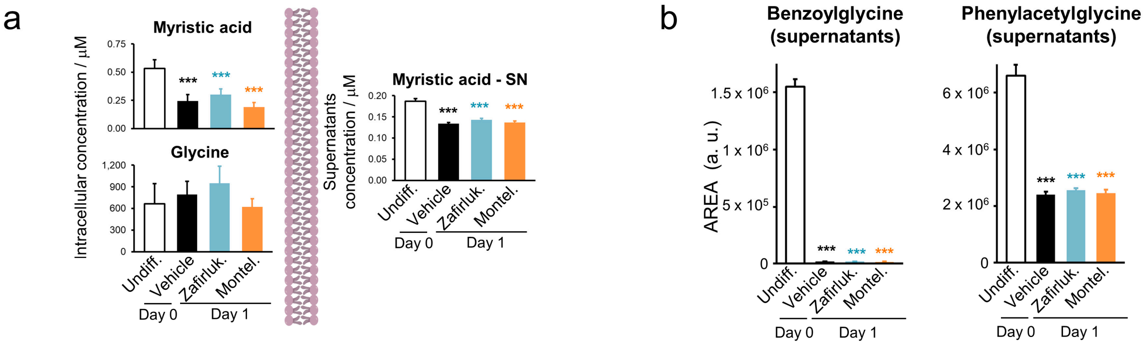

Figure S1. Bar plots of myristoylglycine-related metabolites during SBGS cells differentiation in the presence of zafirlukast and montelukast. (**a**) Absolute quantitation of myristic acid and glycine in SBGS cells in differentiation and their supernatants (myristic acid only). (**b**) Bar plots of glycine conjugated metabolites that were found to be dysregulated in the supernatants of the SGBS cells after 1 day of differentiation. *** $p < 0.001$, one-way ANOVA followed by a Tukey's honestly significant difference *posthoc* test. N = 6 biological replicates per treatment.
